# The Contribution of Polysaccharide Antigens From Clinical *Proteus* spp. and *Klebsiella* spp. Isolates to the Serological Cross-Reactions

**DOI:** 10.3389/fcimb.2021.707578

**Published:** 2021-08-26

**Authors:** Agata Palusiak

**Affiliations:** Department of Biology of Bacteria, Laboratory of General Microbiology, Institute of Microbiology, Biotechnology and Immunology, University of Łódź, Banacha, Poland

**Keywords:** antiserum, cross-reactions, *Klebsiella*, lipopolysaccharide, polysaccharide antigens, *Proteus*

## Abstract

*Klebsiella* spp. and *Proteus* spp. cause hospital-acquired urinary tract infections (UTIs), which are often related to the use of catheters. To create a vaccine preventing UTI, immunogenic bacterial antigens with common epitopes are still being looked for. In this work, the role of polysaccharide antigens of four *Klebsiella* spp. and eight *Proteus* spp. strains in serological cross-reactions with specific antisera was examined. Enzyme-linked immunosorbent assay (ELISA), Western blotting, and silver staining by Tsai method were performed. The *Klebsiella* and *Proteus* spp. LPSs and cells were used as antigens. Polyclonal rabbit sera specific to *Klebsiella oxytoca* 0.023 and 0.062 strains and four *Klebsiella* spp. LPSs were obtained. The ELISA and Western blotting results showed the strongest cross-reactions occurring between lipopolysaccharides (LPSs) from four *Klebsiella* strains and *P. vulgaris* O42 antiserum. The silver-staining procedure revealed the patterns typical of both slow- and fast-migrating mass species of the *Klebsiella* LPSs. The *Klebsiella* spp. antigens also cross-reacted with four *P. penneri* antisera, and most of the reactions were observed as low-migrating patterns. From two *K. oxytoca* antisera obtained in this work, only one, the *K. oxytoca* 0.062 antiserum, cross-reacted with satisfactory strength with *P. penneri* LPSs (19, 22, and 60). Obtaining cross-reactions between the antigens of *Klebsiella* strains and *Proteus* antisera and in the opposite systems is important for proving the immunogenic role of polysaccharide antigens in triggering the immunological response.

## Introduction

*Klebsiella* and *Proteus* genera were described in the same year, 1985 (O’Hara et al., 2000; Brisse et al., 2006). Among *Klebsiella* isolates, *K. pneumoniae* and *K. oxytoca* are predominating as infectious agents (Brisse et al., 2006). Within *Proteus* spp., *P. mirabilis* are the most common cause of infections; however, the isolation frequency of *P. penneri* and *P. vulgaris* has recently been on the increase (Brisse et al., 2006; Palusiak, 2013). These pathogens colonize the human intestinal tract, and in peculiar conditions, they trigger the infections, especially of the urinary tract (UTIs) and wounds as well as bacteremia in immune-compromised patients (O’Hara et al., 2000; Palusiak, 2013; Wu and Li, 2015). *P. mirabilis* and *K. pneumonia* have also an ability to cause coinfections of the urinary tract and, with other uropathogens, coform a biofilm on urinary catheters (Macleod and Stickler, 2007). UTIs caused by both pathogens are often difficult to treat because of the complications, i.e., biofilm development or bacteremia and the increasing multiresistance of the bacteria to antibiotics, especially nitrofurantoin (Macleod and Stickler, 2007; Cuevas et al., 2010; Schaffer and Pearson, 2015; Wu and Li, 2015). This situation requires searching for the ways of protection against the *K. pneumonia* and *P. mirabilis* infections. There are a few available vaccines, which contain the whole cells of different uropathogens including *K. pneumonia* and *P. mirabilis* (Drzewiecka and Lewandowska, 2016). However, the vaccines, e.g., Uromune^®^ should be administered in many doses, which do not result in gaining a long-term protection (Lorenzo-Gómez et al., 2013; Drzewiecka and Lewandowska, 2016). Therefore, scientists are still looking for immunogenic bacterial antigens, which may be used for the construction of a vaccine, e.g., MrpH (a tip adhesion of mannose-resistant *Proteus*-like fimbriae) and *Proteus* toxic agglutinin (Pta) in the case of *P. mirabilis* or capsule polysaccharides (CPS) in the case of *K. pneumonia* (Brisse et al., 2006; Schaffer and Pearson, 2015; Drzewiecka and Lewandowska, 2016). The structural similarities observed for the lipopolysaccharide (LPS) core and the O-polysaccharide (OPS) regions of both species led to a question whether these antigens could contribute to the cross-reactions between *Proteus* antisera and *Klebsiella* spp. cells (Vinogradov et al., 2002; Regué et al., 2005; Knirel et al., 2011). In the previous studies, the reactions of nine *Proteus* antisera and the cells of selected *Klebsiella* spp. clinical isolates were described (Palusiak, 2015). Most of the Western blotting reactions concerned protein antigens. LPS from *K. oxytoca* 0.062 was also proved to be cross-reacting with *Proteus* antisera. In the present study, it was examined if the abovementioned cross-reactions appeared also in the opposite systems: *K. oxytoca* 0.062 antiserum and *Proteus* spp. LPSs. Besides, more cross-reactions between the antigens of four *Klebsiella* spp. strains and *Proteus* antisera were obtained, which is very important for proving the immunogenic role of polysaccharide antigens in triggering the immunological response.

## Material and Methods

### Bacterial Antigens and Sera Obtaining

At the first stage of the study bacterial biomasses were obtained from four *Klebsiella* spp. strains (*K. pneumoniae* 0.08 and *K. oxytoca* 0.023, 0.030, and 0.042) and *P. vulgaris* CCUG 4677 (O42). *Klebsiella* spp. strains are clinical isolates from the urine of patients from the Łódź area provided by the Synevo Laboratory (Łódź, Poland). The tested strains were cultured in nutrient broth supplemented with 0.2% glucose for 18 h at 37°C with aeration. Bacterial cells were centrifuged from the medium (4,845×*g*) and washed two times in distilled water (centrifugation, 4,845×*g*). The sediment was suspended in a small volume of distilled water and subjected to lyophilization. The dry biomasses were suspended in water to a concentration of 2 mg/ml and used as antigens in Western blotting.

In the next step of the research, bacterial LPSs were used as antigens in detailed serological studies. *Proteus* spp. LPSs (*P. penneri* 2 (O66), 22 (O63), and 60 (O70) and *P. vulgaris* CCUG 4677 (O42)) came from the collection of the Laboratory of General Microbiology where they are stored at −20°C. LPSs were extracted from dry biomasses of *K. pneumoniae* 0.08, *K. oxytoca* 0.023, 0.030, and 0.042 strains in 45% phenol for 5 min at 65°C using the phenol-water method, developed by Westphal and Jann (1965) with modifications described by Palusiak (2015). The water phase containing LPS with long OPS chains was collected after centrifugation (4,845×*g*) and the extraction was repeated. Next, the water phases were combined, dialyzed from phenol for 48 h, and centrifuged (4,845×*g*). The LPS solution was adjusted to 2% CH_3_COONa, and a crude LPS was precipitated by the addition of 2 V of 96% ethanol. Finally, the LPS precipitate was dissolved in distilled water, dialyzed (24 h, 4°C), and lyophilized.

LPSs and biomasses used as antigens in SDS-PAGE were purified from proteins by treating the samples, suspended in a loading buffer and boiled for 10 min, with proteinase K (10 mg/ml) for 1 h at 60°C.

The sera specific to the *Proteus* spp. strains tested came from the collection of the Laboratory of General Microbiology whereas the sera specific to *K. oxytoca* (0.023 and 0.062) isolates were obtained by intravenous immunization of a rabbit with bacterial suspension (1.5 × 10^10^ cfu/ml^−1^) at three doses (5-day intervals): 0.25, 0.5, and 1 ml and one dose, 1 ml, after 5 days. The control blood sample was also acquired from a rabbit before vaccination to check by enzyme-linked immunosorbent assay (ELISA) if any *K. oxytoca* spp.-specific antibodies were present in the serum. A protocol was developed to optimize doses and intervals between them to protect the animal from adverse effect on the one hand and to obtain fully reactive serum on the other.

### Serological Studies

Four *Klebsiella* spp. polysaccharides were tested in an ELISA with one of the sera (*K. oxytoca* 0.023) obtained during this work. Fifty nanograms of LPSs was used per well of the F96 Maxisorp Nunc-Immuno plates (12 h, +4°C). To remove uncoated antigens, the plates were washed with water and blocked with casein buffer (2.5% casein, 240 mM of NaOH, 1.5 mM of KH_2_PO_4_, 8 mM of Na_2_HPO_4_, pH 7.2) for 1 h at 37°C. The antisera were serially diluted in a phosphate buffer solution (PBS) starting from the concentration 1:2,000 (1 h at 37°C). As secondary antibodies, rabbit-IgG-specific goat antibodies labeled with horseradish peroxidase (Jackson ImmunoResearch, West Grove, PA, USA) were applied in a dilution of 1:5,000 in casein buffer (50 µl/well; 1 h at 37°C of incubation). The solution of 2,2′-azino-bis(3-ethylbenzothiazoline-6-sulfonic acid) diammonium salt (ABTS) in substrate buffer with the addition of 3% H_2_O_2_ (1:25) was used (50 µl/well) as a substrate for peroxidase (30 min of incubation at 37°C). The addition of oxalic acid (222 mM) stopped the color reaction. The absorbance (A_405_) was measured by using a Multiskan Go microplate reader (Thermo Scientific, Waltham, MA, USA). Antibody titers were determined as the highest antiserum dilution giving an absorbance of ≥0.2.

The basic method used for detecting the cross-reactions between *Proteus* spp. antisera with *Klebsiella* antigens or *K. oxytoca* antisera with *Proteus* spp. LPSs was Western blotting preceded by sodium dodecyl sulphate polyacrylamide gel electrophoresis (SDS PAGE). Of the sample (bacterial biomass or LPS), 8–10 µg was added per lane of polyacrylamide 3% stacking gel and separated during SDS PAGE on 200 V. The samples were transferred to nitrocellulose (Whatman Schleicher & Schuel, Dassel, Germany) in a transfer buffer (25 mM Tris/HCl, 192 mM of glycine, 20% methanol) on 100 V for 1 h. After 1 h of incubation, the nitrocellulose with 10% skimmed milk in a dot-blot buffer (50 mM of Tris/HCl pH 7.4, 200 mM of NaCl), appropriate rabbit antiserum diluted 1:100 in a dot-blot-10% skimmed milk buffer was added (2 h of incubation). The rabbit-IgG-specific goat antibodies conjugated with alkaline phosphatase (Jackson ImmunoResearch) diluted 1:5,000 in a dot-blot-10% skimmed milk buffer were used as secondary antibodies. A color reaction was developed using the AP Conjugate Substrate Kit (Bio-Rad, Hercules, CA, USA). The 3-Color High Prestained Protein Marker (11–245 kDa) DNA GDAŃSK was applied. The blots were scanned by means of Canon Toolbox 4.9.

### Adsorption Procedure

Additionally, to check the similarity between four *Klebsiella* spp. LPSs, the *K. oxytoca* 0.023 antiserum was adsorbed with the biomass of one of each *Klebsiella* spp.-tested strains and checked with these LPSs in Western blotting. The biomasses were diluted 1:100 in PBS and added to the serum in a proportion of 1:10 to remove antibodies specific to the cell antigens. The adsorption was conducted for 30 min in ice and repeated two times (Drzewiecka et al., 2008).

### Gel Staining

To check the electrophoretic patterns of four *Klebsiella* spp. polysaccharide antigens, the polyacrylamide gel with separated samples was prestained with Alcian blue followed by silver staining by the Tsai method (Tsai and Frasch, 1982; Møller and Poulsen, 1995) with modifications described previously by Palusiak (2015). The gel was scanned using Canon’s Toolbox 4.9 software.

## Results

### Determining the Contribution of *Klebsiella* spp. Polysaccharide Antigens to the Reactions of *Proteus* Antisera

**Figure 1:**
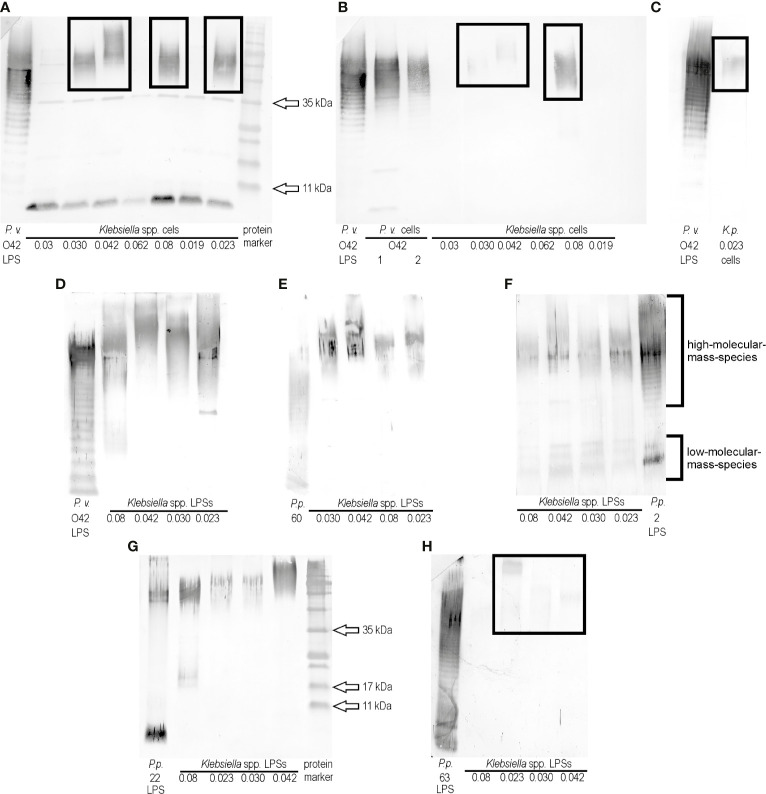
Western blotting reactions of **(A)** *P. vulgaris* O42 antiserum with *Klebsiella* spp. cells, **(B)**, **(C)** *P. vulgaris* O42 antiserum with *Klebsiella* spp. cells treated with proteinase K, **(D)** *P. vulgaris* O42 antiserum, **(E)** *P. penneri* 60 antiserum, **(F)** *P. penneri* 2 antiserum, **(G)** *P. penneri* 22 antiserum, and **(H)** *P. penneri* 63 antiserum with *Klebsiella* spp. LPSs. *Proteus* spp. LPSs homologous to the tested sera were used as the controls of the reactions specificity.

The previous screening of ELISA results for 24 *Klebsiella* spp. strains and polyclonal rabbit antisera specific to the representatives of 79 *Proteus* O serogroups revealed that two *K. oxytoca* strains, 0.030 and 0.042, gave the biggest number of cross-reactions (Palusiak, 2015), thus they both were selected for the present studies. The biomasess of the strains cross-reacted in ELISA to the highest titers (1:256,000 and 1:64,000, respectively) with the serum specific to the *P. vulgaris* 4677 strain (O42) (unpublished data). In the present study, the serum was checked with the selected cross-reacting *Klebsiella* spp. strains in Western blotting using the *P. vulgaris* (O42) LPS as a control. All tested *Klebsiella* spp. biomasses showed the electrophoretic pattern (single bands) characteristic for protein reactions (approximate sizes 35 kDa and below 11 kDa) (**Figure 1A**). Except these reactions, four strains: *K. pneumoniae* 0.08 and *K. oxytoca* 0.023, 0.030, and 0.042 gave the reactions typical of high-molecular-mass polysaccharides species (**Figure 1A**, the reactions are marked with the frames). To confirm that polysaccharides contributed to the observed cross-reactions, the bacterial cells were treated with proteinase K and tested once more in Western blotting with *P. vulgaris* O42-specific serum. The single bands disappeared (**Figures 1B, C** **)**, which confirmed the protein contribution to the previously observed reactions (**Figure 1A**). The low-migrating bands (marked with the frames) were weaker (**Figures 1B, C** **)** than those obtained before the proteinase K treatment of the samples (**Figure 1A**). The obtained results suggest the existence in *P. vulgaris* O42 LPS of an epitope/epitopes common to polysaccharide antigens of the four *Klebsiella* strains tested. To confirm a contribution of LPS to serological cross-reactions, these antigens were obtained from the *Klebsiella* spp. strains by the modified Westphal method (Westphal and Jann, 1965). The LPSs were purified with proteinase K before usage and tested in Western blotting with the serum specific to the *P. vulgaris* O42 (**Figure 1D**). The reactions were stronger than those with the proteinase K-treated cells (**Figures 1B, C** **)** and also concerned the high-molecular-mass species-containing polysaccharides. The reaction of *K. oxytoca* 0.08 LPS distinguished itself from the reactions of other *Klebsiella* LPSs. The remaining LPSs reacted similarly. There were no reactions at the level corresponding to the low-molecular-mass-species containing the core-lipid A fractions (**Figure 1D**).

In the previous study, the cells of *Klebsiella* spp. 0.008, 0.023, 0.030, and 0.042 had been found to cross-react in ELISA (the titer ≥1:32,000) with four *Proteus* antisera: *P. penneri* 2 (O66), 22 (O66), 60 (O70), and 63 (O68) (Palusiak, 2015). To check if the reactions concerned the polysaccharide antigens, in the present work, the *Klebsiella* spp. 0.008, 0.023, 0.030, and 0.042 LPSs were tested in Western blotting with the four abovementioned sera. An appropriate homologous *Proteus* spp. LPS was used as a control of the reaction specificity (**Figures 1E–H**). The strongest reactions were observed for *P. penneri* 60 antiserum at the level corresponding to the high-molecular-mass species-containing polysaccharides (**Figure 1E**). The strong reactions were also obtained for *P. penneri* 2 antiserum, and they concerned both high- and low-molecular-mass species corresponding to polysaccharides and core-lipid A fractions, respectively (**Figure 1F**). The reactions of *P. penneri* 22 antiserum with *Klebsiella* spp. 0.042 and 0.08 antigens were stronger than those with 0.030 and 0.023 LPSs (both presented similar low-migrating bands) (**Figure 1G**). The weakest low-migrating patterns appeared in the reactions (marked with the frame) of the *P. penneri* 63 antiserum with three *Klebsiella* spp. LPSs (**Figure 1H**).

### Determining the Contribution of *Proteus* spp. Lipopolysaccharides to the Reactions of *K. oxytoca* Antisera

**Figure 2:**
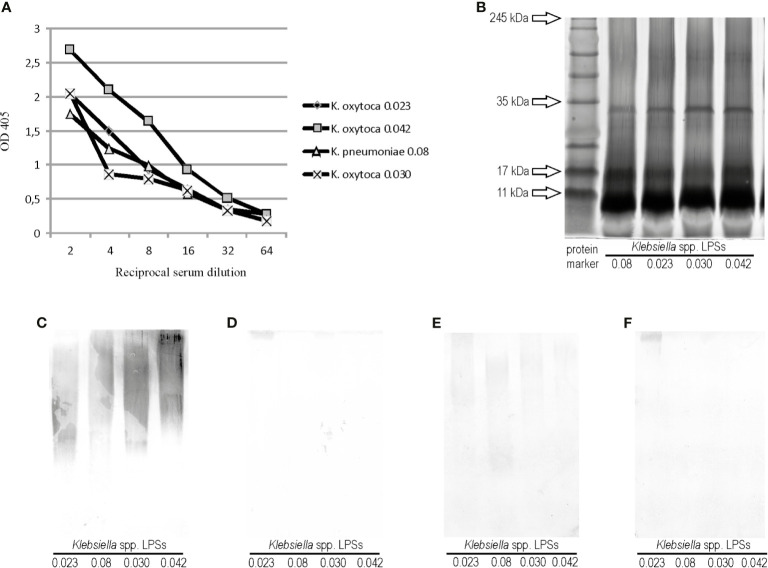
**(A)** Reactivity of *K. oxytoca* 0.023 serum with *Klebsiella* spp. tested LPSs in ELISA. **(B)** SDS-PAGE and Alcian blue-silver staining procedures obtained for *Klebsiella* spp. LPSs tested. Lane 1 shows prestained protein marker; Western blotting reactions of **(C)** *K. oxytoca* 0.023 unadsorbed serum (control), **(D)** *K*. *oxytoca* 0.023 serum adsorbed by the *K. pneumoniae* 0.08 cells, **(E)** *K. oxytoca* 0.023 serum adsorbed by the *K. pneumoniae* 0.042 cells, and **(F)** *K. oxytoca* 0.023 serum adsorbed by the *K. pneumoniae* 0.030 cells with *Klebsiella* spp.-tested LPSs.

The polyclonal rabbit serum specific to one of the four cross-reacting *Klebsiella* strains, *Klebsiella oxytoca* 0.023, was obtained to check if the cross-reactions also appeared in the opposite systems: *Proteus* LPSs and *Klebsiella* antisera. The *Klebsiella oxytoca* 0.023 antiserum was tested in ELISA and Western blotting with *Klebsiella* LPSs, 0.08, 0,030, and 0,042, to see if they showed any serological similarities to each other and to the homologous LPS (**Figures 2A, C** **)**. In ELISA, all *Klebsiella* spp. antigens reacted to the same antiserum titer (1:64,000), but the antibody binding curve obtained for the reaction with *K. oxytoca* 0.042 LPS differed from the other curves (**Figure 2A**). After silver staining, the electrophoresis pathways of the tested *Klebsiella* LPSs were found to be very similar to each other (**Figure 2B**). The silver-staining procedure revealed the patterns typical of both slow- and fast-migrating LPS mass species. In Western blotting, the serum reactions with the four *Klebsiella* spp. LPSs tested were very similar and concerned only the high-molecular mass species of polysaccharides (**Figure 2C**). To verify the assumption on the serological similarities of the tested *Klebsiella* antigens, the *K. oxytoca* 0.023 serum was adsorbed three times with the cells of the appropriate *Klebsiella* spp. strains (0.08, 0.030, or 0.042) and checked once more with all the four tested LPSs in Western blotting. The reactions were almost completely abolished after the adsorption of the serum with the cells of *K. pneumoniae* 0.08 and *K. oxytoca* 0.030 (**Figures 2D, F** **)**, which is a result of removing the cross-reacting antibodies from the serum. The adsorption of the serum with *K. oxytoca* 0.042 cells left the antibodies weakly recognizing epitopes in the 0.08, 0.023, and 0.030 polysaccharides. However, the intensity of these reactions was significantly weaker (**Figure 2E**) compared with the strength of reactions in a control (unadsorbed serum) (**Figure 2C**). Complete or almost complete abolition of the reactions of adsorbed *K. oxytoca* 0.023 antiserum with the tested *Klebsiella* LPSs suggests that polysaccharide antigens of the four *Klebsiella* strains are serologically very similar. The antigen, which appeared to be the most serologically different from the tested *Klebsiella* spp. LPSs was *K. oxytoca* 0.042.

**Figure 3:**
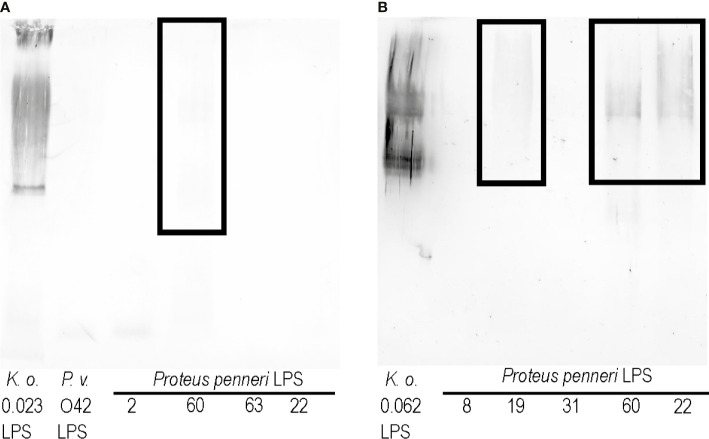
Western blotting reactions of **(A)** *K. oxytoca* 0.023 serum and **(B)** *K. oxytoca* 0.062 serum with *Proteus* spp. LPSs.

As shown in **Figures 1D–H**, the *K. oxytoca* 0.023 LPS cross-reacted with the sera specific to the *P. penneri* 2, 22, 60, and 63 strains and *P. vulgaris* O42 strain. To see if cross-reactions also appeared in the opposite system, the *Proteus* spp. LPSs were checked in Western blotting with the *K. oxytoca* 0.023 antiserum (**Figure 3A**). Among the tested LPSs, only *P. penneri* 60 LPS very weakly cross-reacted at the level corresponding to high-molecular-mass species containing LPS with O-polysaccharide chains (**Figure 3A**, the reaction is in the frame).

To see if it is possible to obtain more cross-reactions of *Klebsiella* antisera and *Proteus* LPSs, another serum, specific to the *K. oxytoca* 0.062 strain was obtained. As previously revealed, the LPS obtained from this strain strongly cross-reacted with sera specific to *P. penneri* 8, 19, 31, 60, and 22 (Palusiak, 2015). In the present study, the *K. oxytoca* 0.062 antiserum was tested with all the abovementioned LPSs in Western blotting. Cross-reactions were observed for high-molecular-mass species containing LPS with O-polysaccharide chains of three LPSs: *P. penneri* 19, 22, and 60 (marked in **Figure 3B** with the frame). The reactions obtained for *P. penneri* 22 and 60 LPSs were the strongest.

## Discussion

**Figure 4:**
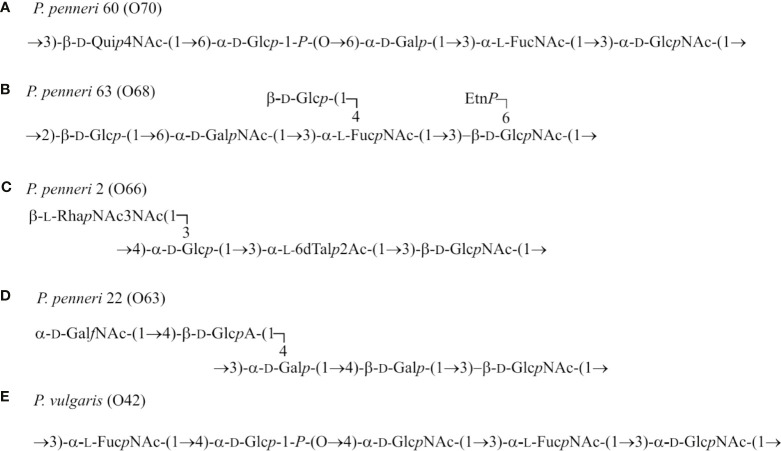
Structures of the LPS O-polysaccharides of **(A)** *P. penne*ri 60 (O70), **(B)** *P. penneri* 63 (O68), **(C)** *P. penneri* 2 (O66), **(D)** *P. penneri* 22 (O63), and **(E)** *P. vulgaris* (O42) (Knirel et al., 2011).

*K. pneumoniae* and *P. mirabilis* are common Gram-negative opportunistic pathogens causing nosocomial infections, among which urinary tract infections have been reported to be the most frequent (O’Hara et al., 2000; Brisse et al., 2006; Macleod and Stickler, 2007). For both species, increasing numbers of multidrug resistant (MDR) strains have been recently identified (Cuevas et al., 2010; Schaffer and Pearson, 2015; Wu and Li, 2015; Drzewiecka and Lewandowska, 2016). The presence of the MDR phenotype may result in recurrent and difficult-to-treat infections. What is more, some antibiotics may increase the release from bacterial cells of LPS—endotoxin, playing a crucial role in septic shock development. This devastating state results from a cascade of immune system activities occurring in response to large amounts of LPS particles in the bloodstream. A complex of LPS and LPS-binding protein (LPB) binds to the CD14 receptor, located on macrophages, monocytes, or neutrophils, and then associates with the Toll-like receptor (TLR) 4 containing a domain required for signal transfer. This leads to the activation of the transcription factors and release of the tumor necrosis factor α, interleukins (IL-1, IL-6, IL-8, IL-12), nitric oxide, prostaglandins, leukotrienes, or the platelet-activating factor by macrophages. Overproduction of mediators causes fever, tachycardia, leucopenia, and vascoconstriction which may result in organ damage or even death of a patient with sepsis (Müller-Loennies et al., 2007). Facing this alarming situation, a need arises to find an alternative way of prevention from the abovementioned infections caused by *Klebsiella* and *Proteus* spp. *bacilli*. The first step of research should include searching for an antigenic relationship between the bacterial clinical strains. As far as *Proteus* spp. strains are concerned, a lot of studies have been performed on the serospecificity and structures of poly- and oligosaccharide parts of LPSs, which has resulted in forming the schemes of strains’ classification into O and R serotypes (Knirel et al., 2011; Palusiak, 2016). As for *Klebsiella* spp. strains, the best structurally and serologically characterized polysaccharide is capsular polysaccharide (CPS), which is the most outstanding part of the bacterial cell (Wu and Li, 2015; Brisse et al., 2006). The cross-reactions had been previously observed for the antigens of each genus, but they concerned the reactions between: *Proteus* LPS and heterologous *Proteus* antisera or *Klebsiella* spp. CPS and anti CPS *Klebsiella* sera (Brisse et al., 2006; Knirel et al., 2011; Knirel, 2011). In the first study regarding the search for cross-reacting antigens of *Klebsiella* spp. and *Proteus* spp. strains, a lot of cross-reactions were described, some of which were typical for polysaccharides (Palusiak, 2015). In this work, other LPSs: from three *K. oxytoca* 0.023, 0.030, and 0.042 strains and one *K. pneumoniae* 0.08 strain were serologically characterized. This time cross-reactions of the mentioned *Klebsiella* spp. antigens were observed for five *Proteus* spp. antisera (**Figures 1D–H**). In the case of four sera, specific to *P. penneri* 22, 60, and 63 and *P. vulgaris* O42 strains, the reactions concerned only the high-molecular-mass species of polysaccharides (**Figures 1D, E, G, H** **)**. In the tested *Proteus* spp. antisera, the O-polysaccharide-specific antibodies dominated since all strains homologous to the sera presented the smooth form of LPSs (Knirel et al., 2011). The observed cross-reactions may suggest the existence of common epitopes in the tested polysaccharide antigens. Each of the cross-reacting *Proteus* spp. LPSs presents a different O-serotype, but some of them share a common fragment. While analyzing the O-antigen structures (Knirel et al., 2011) of the cross-reacting *Proteus* spp. LPSs, it can be observed that α-l-FucNAc-(1→3)-d-Glc*p*NAc(1- are found in OPSs of the three strains: *P. penneri* 60 (O70), 63 (O68), and *P. vulgaris* O42 (**Figure 4**). Contribution of the sera specific to the three *Proteus* spp. LPSs to the cross-reactions and the fact that these antigens share a common fragment of OPS suggest a potential role of this disaccharide as one of the epitopes.

In the present study, the *P. penneri* 2 antiserum cross-reacted with the tested *Klebsiella* spp. LPSs at the level corresponding to the low-molecular fraction of the core-lipid A, which were found to be very similar to the corresponding band obtained for *P. penneri* 2 LPS. It might indicate the presence of common epitopes in the core regions of all tested LPSs (**Figure 1F**). It is likely since the LPS core regions of *K. pneumoniae* and *Proteus* spp. strains are very similar in their structures, especially in the inner part (Vinogradov et al., 2002; Regué et al., 2005; Palusiak, 2016).

The novel aspect of this work concerns obtaining *K. oxytoca* 0.023 and 0.062 antisera and testing them in Western blotting with respective *Proteus* spp. LPSs. For the *K. oxytoca* 0.023 antiserum, a very slight reaction was observed with *P. penneri* 60 LPS, and it concerned high-molecular-mass species (**Figure 3A**), but the strength of the reaction was not satisfactory. **Figure 3A** shows how difficult it is to obtain cross-reactions in opposite systems: anti-*Klebsiella* sera and *Proteus* spp. LPSs. In the case of the *K. oxytoca* 0.062 antiserum, the results were more promising. This time cross-reactions were observed with three out of five LPSs tested, *P. penneri* LPSs, 19, 22, and 60, at the level corresponding to high-molecular-mass species containing LPS with O-polysaccharide chains; however, the reactions were much weaker than those which had been previously observed with *K. oxytoca* 0.062 LPS and *Proteus* antisera (Palusiak, 2015). It is worth emphasizing that *P. penneri* 60 and 22 LPSs weakly cross-reacted with the *K. oxytoca* 0.062 antiserum (**Figure 3B**). What is more, the stronger cross-reactions could be seen in opposite systems: *K. oxytoca* 0.062 LPS with the *P. penneri* 22 and 60 antisera (previous study, Palusiak, 2015). Confirmation of the contribution of the mentioned antigens to the cross-reactions in both a combination of the antigens and the sera may indicate them as potential antigens for future studies on a polyvalent vaccine, which could induce the cross-protection against *Klebsiella* spp. and *Proteus* spp. infections. The immunogenic antigens like polysaccharide fragments common to the numerous bacterial strains used for the conjugate vaccine construction may be more effective than whole bacterial cells in triggering the specific response. The native LPS cannot be used for a vaccination due to its toxicity. To eliminate this effect, LPS should be lacking in lipid A—a biological center of LPS toxicity or should be incorporated to liposomes (Poxton, 2004). Such vaccines (e.g., a liposomal vaccine containing the complete-core LPSs of *Escherichia coli* K-12, R1, *Pseudomonas aeruginosa* PAC608, and *Bacteroides fragilis* or a conjugated vaccine including a conjugate of the *E. coli* R1core region with tetanus toxoid) tested on an animal model appeared to be nontoxic and broadly immunogenic (Ługowski et al., 1996; Bennett-Guerrero et al., 2000). As for using the LPS vaccine in human, the clinical studies by Launay et al. showed that the candidate *Shigella sonnei* vaccine with antigen O (1790GAHB) administrated to healthy European adults (18–45 years old) was well tolerated and did not cause serious adverse effects (Launay et al., 2017). It should be remembered that the subunit vaccine formula requires an addition of an adjuvant, which boosts the antigens immunogenicity. The monophosphoryl lipid A (MPL) adjuvant containing modified *Salmonella minnesota* lipid A is a component of vaccines registered for use in Europe (FENDrix), which is another example showing the significance of the studies on LPS (Casella and Mitchell, 2008).

It is worth mentioning that most *Klebsiella* strains tested in this work belong to *K. oxytoca* species, not as well known as *K. pneumoniae* but also often isolated from clinical materials (Brisse et al., 2006) and the serological data presented in this paper expand the knowledge on the species.

## Data Availability Statement

The raw data supporting the conclusions of this article will be made available by the authors, without undue reservation.

## Ethics Statement

The animal study was reviewed and approved by Local Ethic Committee for Animal Testing from Łódź.

## Author Contributions

The author confirms being the sole contributor of this work and has approved it for publication.

## Funding

This study was supported by the grant for the young scientists and doctoral students, No. B1311000000061.02, from the University of Łódź and financed by Ministry of Sciences and Higher Education (Poland).

## Conflict of Interest

The author declares that the research was conducted in the absence of any commercial or financial relationships that could be construed as a potential conflict of interest.

## Publisher’s Note

All claims expressed in this article are solely those of the authors and do not necessarily represent those of their affiliated organizations, or those of the publisher, the editors and the reviewers. Any product that may be evaluated in this article, or claim that may be made by its manufacturer, is not guaranteed or endorsed by the publisher.

## Abbreviations

CPS, capsular polysaccharide; ELISA, enzyme-linked immunosorbent assay; Gal, galactose; Glc, glucose; GlcA, glucuronic acid; GlcNAc, 2-acetamido-2-deoxy-glucose; LPB, LPS-binding protein; LPS, lipopolysaccharide; MDR, multidrug resistant; MPL, monophosphoryl lipid A; OPS, O-polysaccharide; PBS, phosphate buffer solution; Rha, ramnose; SDS PAGE, sodium dodecyl sulphate polyacrylamide gel electrophoresis; TLR, toll-like receptor; UTIs, urinary tract infections.
